# Sequencing and validation of housekeeping genes for quantitative real-time PCR during the gonadotrophic cycle of *Diploptera punctata*

**DOI:** 10.1186/1756-0500-6-237

**Published:** 2013-06-19

**Authors:** Elisabeth Marchal, Ekaterina F Hult, Juan Huang, Stephen S Tobe

**Affiliations:** 1 Department of Cell and Systems Biology, University of Toronto, 25 Harbord Street, Toronto, Canada

**Keywords:** q-RT-PCR, Reference genes, GeNorm, Normfinder, Normalization, Diploptera punctata

## Abstract

**Background:**

Quantitative RT-PCR (q-RT-PCR) is a powerful tool that allows for the large scale analysis of small changes in gene expression. Accurate and reliable results depend on the use of stable reference genes for normalization. However, the expression of some widely used housekeeping genes can vary under different experimental setups. To our knowledge, no validation studies have been reported for reference genes in cockroaches. The aim of the current study is the identification and validation of a set of eight housekeeping genes during the first gonadotrophic cycle of the cockroach, *Diploptera punctata*. This study made use of two different algorithms (geNorm and Normfinder) to evaluate the stability of gene expression.

**Results:**

Candidate housekeeping genes were sequenced: *β-actin* (*Actin*), *elongation factor 1 alpha* (*EF1a*), *glyceraldehyde-3-phosphate dehydrogenase* (*GAPDH*), *armadillo* (*Arm*), *ribosomal protein L32* (*RpL32*), *succinate dehydrogenase* (*SDHa*), *annexin IX* (*AnnIX*) and *α-tubulin* (*Tub*). The expression of these eight genes was analyzed in corpora allata (CA) and ovaries of adult female *D. punctata*. Both geNorm, as well as Normfinder characterized *SDHa*, *EF1a* and *Arm* as being the most stably expressed in the corpora allata. In the ovary, the geNorm calculation showed *Tub*, *EF1a* and *RpL32* to be most stable, whereas Normfinder identified *Tub*, *EF1a* and *Arm* as the best. In ovary, the least stable gene was *Actin*, challenging its usefulness in normalization. As a proof of principle, the expression of *follicle cell protein 3c* and *CYP15A1* was monitored during the first gonadotrophic cycle.

**Conclusion:**

*Arm* and *EF1a* form the most stably expressed combination of two reference genes out of the eight candidates that were tested in the corpora allata. Our results show that the combined use of *Tub*, *EF1a* and *RpL32* ensures an accurate normalization of gene expression levels in ovary of *D. punctata.* Our study has indicated that neither *Actin* nor *AnnIX* should be used for normalization of transcript levels when studying the first gonadotrophic cycle in CA or ovary of *D. punctata*. The results stress the necessity for validation of reference genes in q-RT-PCR studies in cockroaches.

## Background

Given its very high sensitivity, q-RT-PCR is currently the method of choice to measure mRNA transcription levels for individual genes. Results of high-throughput techniques such as microarrays are often confirmed using this robust technique. Moreover, this assay is widely used for medical diagnostics purposes and in identification of disease-specific biomarkers [1-3]. However, the quality of the results depends on the use of the appropriate controls. The calculation of relative transcript levels requires normalization that will compensate for small differences in initial sample quantity, sample preparation and efficiency of cDNA synthesis. However, the use of unpredictable normalization factors can pose difficulties in using q-RT-PCR to accurately describe relative mRNA levels. A common method to normalize mRNA levels of genes of interest is the use of endogenous control genes, termed ‘housekeeping’ genes or reference genes. Ideally, the mRNA levels of these genes should be stable in all cell types and tissues and should not be regulated by internal or external influences in the experimental setup. Since literature suggests that the expression of commonly used reference genes can change in different experimental conditions or tissues and no universally valid reference gene exists, the choice of reference genes cannot be made *a priori *[4-8]. For this reason, several algorithms were developed to identify the most stably expressed references genes in any given experimental setup [4,6,9]. Accordingly, many studies aimed at finding the most suitable reference genes in a diverse array of cells, tissues, experimental treatments and different species are now being reported.

Although there have been several validation studies for reference genes in different invertebrate species [10-16], many expression studies in insects, however, still use only one reference gene and most expression studies in cockroaches have routinely used *ß-actin*. Although limited sequence information is available on the more basal insect order Dictyoptera, the cockroach *Diploptera punctata* has proven to be an ideal model organism in the study of juvenile hormone (JH). JHs represent a family of sesquiterpenoid hormones synthesized in the CA, a pair of small endocrine organs located in the insect’s head. JHs play important and diverse roles during insect life; for example, they are involved in the control of development, growth, aging, metamorphosis and reproduction [17,18]. *D. punctata* is the only known truly viviparous cockroach. Because of this remarkable feature, the rate of JH production is tightly controlled during the animal’s reproductive cycle. High rates of JH biosynthesis in their CA are coordinated with a very precise and predictable order of reproductive events, making this cockroach an ideal model for studying the regulation of JH production [19-21]. Therefore, *D. punctata* has also been used in several studies aimed at identifying novel Insect Growth Regulators (IGRs) that can interfere with JH production [22-25]. Understanding different patterns of gene expression is essential in identifying factors that are important for biosynthesis, degradation, regulation and function of this crucial insect hormone.

The objective of the current study was to evaluate the optimal reference genes for use in normalization of gene transcript levels in CA and ovaries during the gonadotrophic cycle of *D. punctata*. Eight candidate reference genes were sequenced from embryo or adult ovary of *D. punctata*. Their expression levels and stability were measured in two tissues undergoing important cross-talk during the gonadotrophic cycle: the ovary and the CA. Two different algorithms were used to calculate the most stable reference genes in our setup. As a proof of principle, the relative mRNA quantity of two target genes was studied. A gene involved in choriogenesis (*follicle cell protein 3c*) was studied in the ovary and expression of *CYP15A1*, a gene encoding a cytochrome P450 enzyme directly involved in the JH biosynthetic pathway was determined in the CA [26,27].

## Results

### Physiological measurements of the sample animals

**Figure 1:**
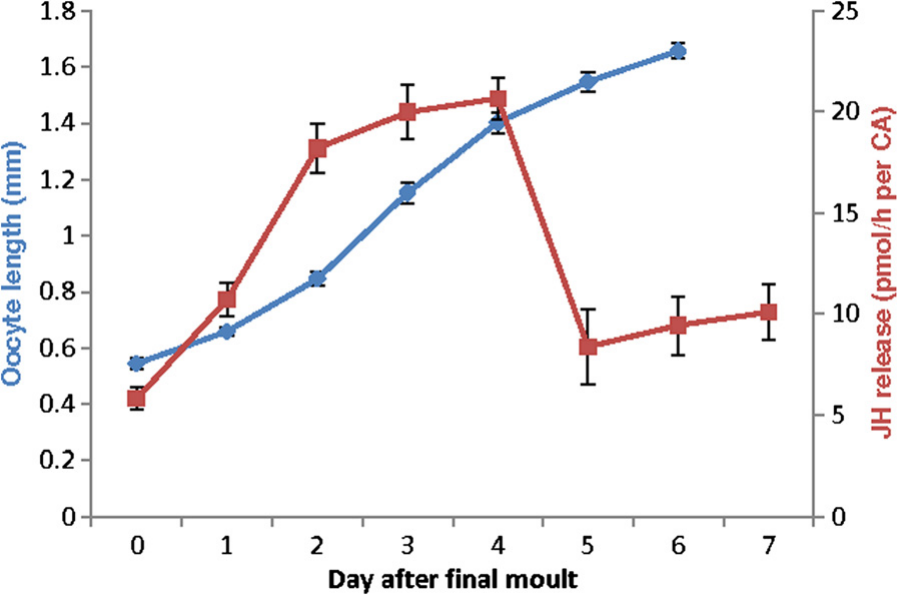
**JH and oocyte measurements of the test animals.** The right-hand axis shows the length of basal oocytes dissected from *D. punctata* females during the first gonadotrophic cycle (day 0 to day 6 after the imaginal molt, oviposition took place at the start of day 7). The data represent means of 30 individual animals. Vertical bars indicate standard deviations. The left-hand axis represents the amount of JH released during the first gonadotrophic cycle (day 0 to day 7 after final molt). The data are represented in pmol/hour per individual CA and represent means of 12 independent biological replicates. The vertical bars indicate standard errors.

Oocyte length was determined for each dissected female cockroach used in the q-RT-PCR assays (Figure 1). A clear rising trend in length can be observed as vitellogenin is taken into the developing oocytes, as was previously described [28]. The sample animals ovulated at the start of day 7. Rates of JH release throughout the gonadotrophic cycle steadily rise until four days post final molt, a sharp decline can be seen on day 5 (Figure 1). The activity of the CA is linked to the cycle of egg development, as was reported by Tobe and Stay [21]: Rates of JH release increase as oocytes grow during vitellogenesis. Before oviposition, JH release decreases and remains at a low level during gestation.

### Sequencing of the candidate reference genes and target gene *follicle cell protein 3c (fcp 3c)*

Partial sequences for eight new candidate reference genes and the target gene *fcp 3c* were cloned in *D. punctata*. PCR amplicons ranging in size from 270 to 1319 bp were generated from *D. punctata* ovary or embryo cDNA templates (see Additional file 1). Upon blastx National Center for Biotechnology Information (NCBI) database searches, the orthologs showed substantial similarity to the corresponding previously described reference genes in other insect species. The nine obtained sequences (*Actin*, *EF1a*, *GAPDH*, *Arm*, *RpL32*, *SDHa*, *AnnIX, Tub* and *fcp 3c*) were uploaded on NCBI’s GenBank and accession numbers were assigned (Table 1). The sequence of the target gene *CYP15A1* was obtained from GenBank [GenBank: AY509244].

**Table 1:** Name, symbol, function, primer sequences and accession number for the housekeeping genes and the target gene

**Name**	**Symbol**	**Function**	**Degenerate primer sequences**	**Accession number (GenBank)**
Actin 5C	Actin	Cytoskeletal structural protein	F 5'-CATCAGGGAGTCATGGTCGGCA-3'	JQ086312
			R 5'-GCATACAGGTCTTTACGGATGTCC-3'	
Elongation factor 1 alpha	EF1-a	Protein synthesis	F 5'-TTCGAGAAGGAAGCCCAGGARATGG-3'	JQ086311
			R 5'-CACDGAYGGACCAATCAGCACAYCT-3'	
Glyceraldehyde-3-phosphate dehydrogenase	GAPDH	Glycolysis	F 5'-ATGTCDAAGATYGGWATCAACGG-3'	KC149901
			R 5'-TTAGTCYTTKGACTGCATG-3'	
Armadillo	Arm	Cell adhesion	F 5'-ATGGARGARATHGTNGARGGAAC-3'	KC149902
			R 5'-TTGTARTCYTGNGGYTTRTCYTC-3'	
Ribosomal protein L32	RpL32	Ribosomal structural constituent	F 5'-AARCCNAARGGNATYGACAAYAG-3'	KC149903
			R 5'-CRCARTAYTTSCKRTTMTGCATCAT-3'	
Succinate dehydrogenase	SDHa	Energy metabolism	F 5'-TGGCAGTGGCAYATGTAYGAY-3'	KC149904
			R 5'-GTYTGCATNGTYTTYTGCAT-3'	
Annexin IX	AnnIX	Cell differentiation, membrane fusion	F 5'-AARTGYACYCCHACRGTRTAYCC-3'	KC149905
			R 5'-TTRATRTCMSCYARRTCNATYTC-3'	
a-tubulin	Tub	Cytoskeletal structural protein	F 5'-ATGCGTGARTGTATHTCRRTBCA-3'	KC149906
			R 5'-GARTCCATNCCNACYTCYTCRTA-3'	
Follicle cell protein 3c	Fcp 3c	Vitellogenesis	F 5'-TGYRYHTGTGGARTGTTYTTRA-3'	KC149907
			R 5'-TCYTTRCARCARTAYTCYCKWCC-3'	

### Q-RT-PCR assays

The initial screening of the eight potential reference genes showed that all of the genes were expressed in both CA and ovary of *D. punctata*. The primer efficiency (E) for each of the eight candidate genes was calculated using the slope acquired when measuring the fluorescence in 10-fold cDNA dilutions of a calibrator cDNA sample using the formula: E = 10^1/-slope^-1. Only primers that fell in the range 90 – 100% efficiency and showed r^2^ > 0.95 were considered for further analysis. The calculated efficiencies ranged from 90.8% (for *AnnIX*) to 100% (for *GAPDH*), the regression coefficients (R^2^) varied from 0.975 (*SDHa*) to 0.995 (*RpL32*) (Table 2). No amplification of the fluorescent signal was detected in the negative control samples (−RT), proving that the RNA extraction methods and the DNase treatment procedures effectively eliminated genomic DNA from the RNA samples. Single amplicons were detected on 2% agarose gels of selected samples for each primer pair after the q-RT-PCR protocol was run. These amplicons were cut out, gel-extracted, subcloned and sequenced, thereby confirming target specificity.

**Table 2:** **q-RT-PCR primer sequences, amplicon size, reaction efficiencies and correlation coefficients in q-RT-PCR assay for the selected references genes and the target genes *follicle cell protein 3c *and *CYP15A1***

**q-RT-PCR primers**	**Forward**	**Reverse**	**Amplicon size (bp)**	**Efficiency (%)**	**R**^**2**^
**Actin**	5'-TCGCACACAGTACCAATCTATGAA-3'	5'-CAAGTCACGACCAGCCAGATC-3'	78	99.3	0.980
**EF1-a**	5'-TCGTCTTCCTCTGCAGGATGTCT-3'	5'-GGGTGCAAATGTCACAACCATACC-3'	109	99.2	0.994
**GAPDH**	5'-TCCGTGTGCCTGTTCCTAATG-3'	5'-GCTGCCTTGCCAAGTCTGA-3'	61	100	0.988
**Arm**	5'-GCTACTGCACCACTCACAGAATTATT-3'	5'-CTGCAGCATACGTTGCAACA-3'	64	94.5	0.980
**RpL32**	5'-GCGCTTCAAGGGCCAGTAC-3'	5'-TGCTTGGTTTTCTTATTGCTACCA-3'	63	97.4	0.995
**SDHa**	5'-GCTCTTCTGTGCATGGTGCTAA-3'	5'-GCACGTCCGAACACAACAAG-3'	70	94.6	0.975
**AnnIX**	5'-CAGAGGTTGGAGATTGCTGA-3'	5'-TGCATCTTCAAATGCTCCTC-3'	96	90.8	0.989
**Tub**	5'-AAATTACCAACGCTTGCTTTGAA-3'	5'-TGGCGAGGATCGCATTTT-3'	58	95.1	0.993
**Fcp 3c**	5'-TCTACCTTGCAACGCTTTTG-3'	5'-TCCCTGTCTATTGAGCCACA-3'	111	92.9	0.975
**CYP15A1**	5'-GTTGGGATCTCGGAGCATGG -3'	5'-CGAACACGTCATGCATCGGT -3'	116	100	0.992

#### Expression stability of the selected reference genes

**Figure 2:**
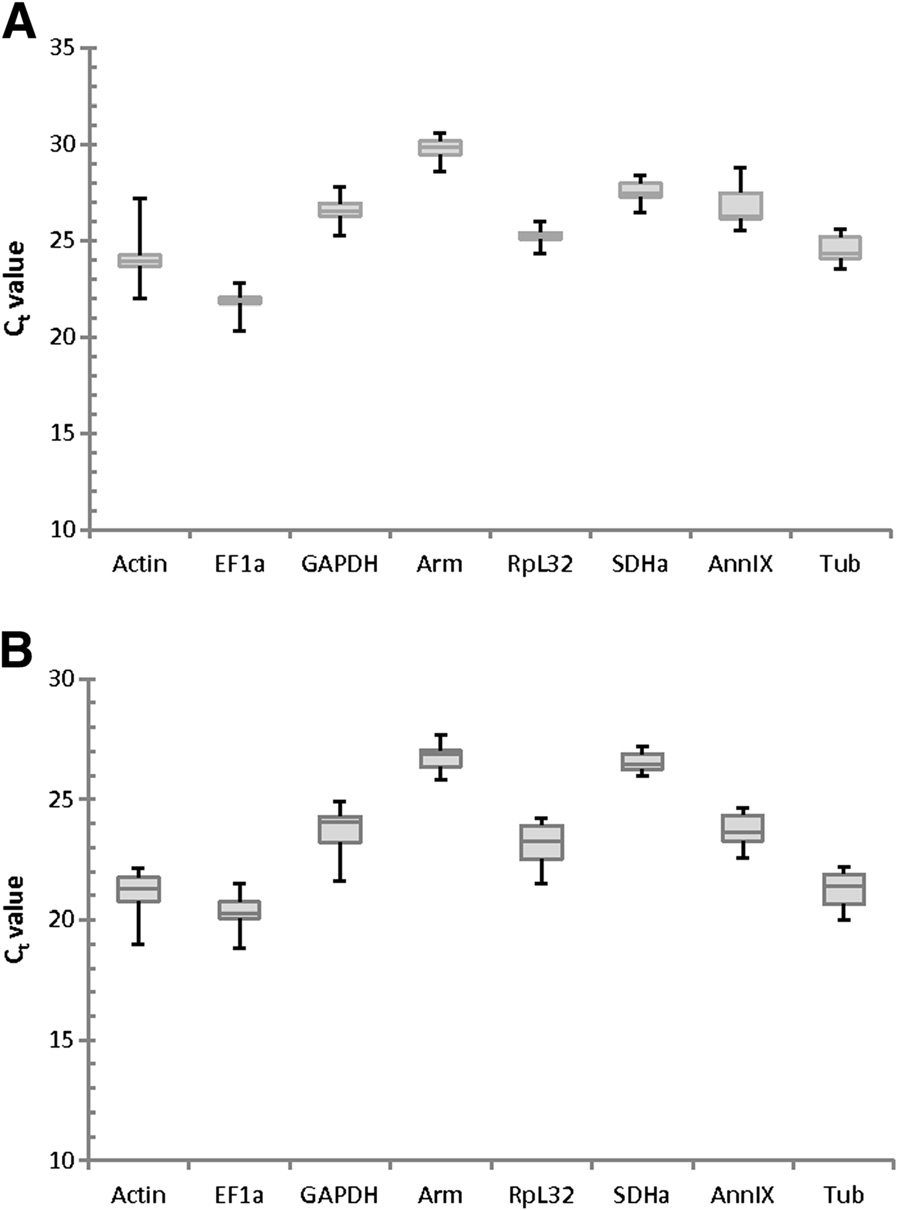
**Boxplots showing the Ct variation of the reference genes.** The variation in Ct values of the eight candidate reference genes in **(A)** the CA samples (3 biological replicates for each time point, n = 24) and in **(B)** the ovary (3 biological replicates for each time point, n = 24) as indicated by the raw Ct values. The values are given as the cycle threshold (Ct, mean of triplicate samples). The boxplots show the 25th quartile, median and the 75th quartile (horizontal lines) and the minimal and maximal values (whiskers).

Variation of the Ct values among the 24 samples for the eight candidate reference genes is shown in Figure 2. Raw Ct values are given in Additional file 2. In the CA, the Ct values for the selected reference genes ranged from 20.33 (*EF1a*) to 30.62 (*Arm*). In the ovary, the Ct values ranged from 18.65 (*EF1a*) to 28.23 (*Arm*). Transcripts for *EF1a* and *Arm* were shown to be the most and least abundant expressions respectively in both tissue types tested. The Ct values between CA and ovary can, however, not be compared since the amount of RNA used to transcribe to cDNA was not the same.

Two different algorithms, GeNorm and Normfinder, were used to identify the genes that were most stably expressed in ovary and CA during the differing physiological conditions of the first gonadotrophic cycle.

**Figure 3:**
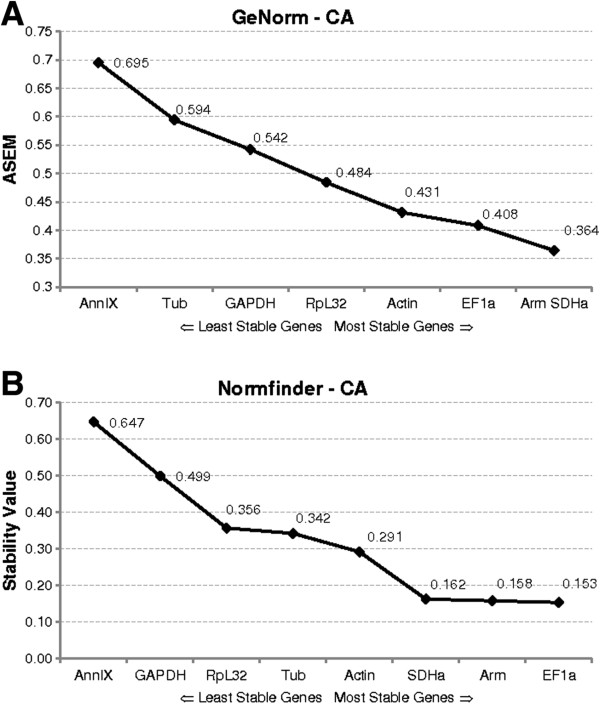
**Stability values in the CA as generated by the two algorithms, A) geNorm and B) Normfinder. (A)** The average expression stability values (AESM) from least stable (left) to most stable (right). Threshold for an unstable gene is M ≥ 1.5. **(B)** The expression stability values from the candidate reference genes calculated by the Normfinder software.

**Figure 4:**
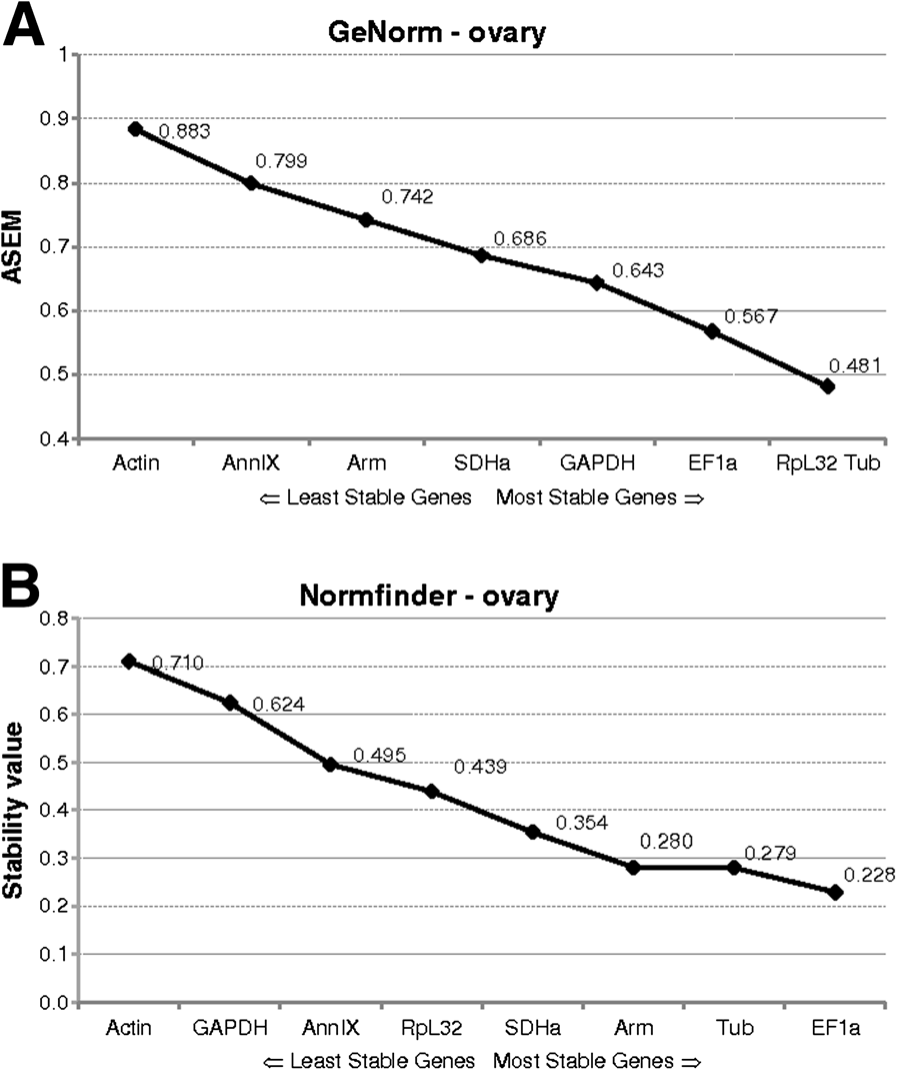
**Stability values in the ovary as generated by the two algorithms, A) geNorm and B) Normfinder. (A)** The average expression stability measure (AESM) from least stable (left) to most stable (right). Threshold for an unstable gene is M ≥ 1.5. **(B)** The expression stability values from the candidate reference genes calculated by the Normfinder software.

The geNorm algorithm assumes that the candidate genes are not co-regulated. For each reference gene tested, geNorm calculates the gene expression stability value M as the average pairwise variation for that gene with all other reference genes. Genes characterised by a low pairwise variation M will therefore have stable expression. The gene with the highest M value will be eliminated and this process is repeated until the two most stable genes are identified. This last pair of genes is recommended as the optimal pair of reference genes. The designers of the geNorm software recommend the use of at least two reference genes to ensure accurate normalization. Figures 3A and 4A show the average expression stability measures (AESM) of the set of reference genes tested in the CA and ovary respectively.

In the CA, geNorm ranked the suite of candidate reference genes: *Arm*, *SDHa* >*EF1a* >*Actin* >*RpL32* > *GAPDH* >*Tub* >*AnnIX*. *Arm* and *SDHa* were found to be the most stable genes with an average expression stability M of 0.364. The gene with the least stable expression was *AnnIX* with an M-value of 0.695. In the ovary, use of the geNorm software provided the following ranking: *RpL32*, *Tub* >*EF1a* >*GAPDH* >*SDHa* >*Arm* >*AnnIX* >*Actin*. *RpL32* and *Tub* were found to be the best genes to ensure accurate normalization with an M value of 0.481, and least stable was *Actin* (M = 0.883). All genes were found to be sufficiently stable for use in normalization. GeNorm sets a threshold for an unstable gene as an AESM of 1.5, well above our maximal observed value of 0.695 and 0.883 in CA and ovary respectively.

**Figure 5:**
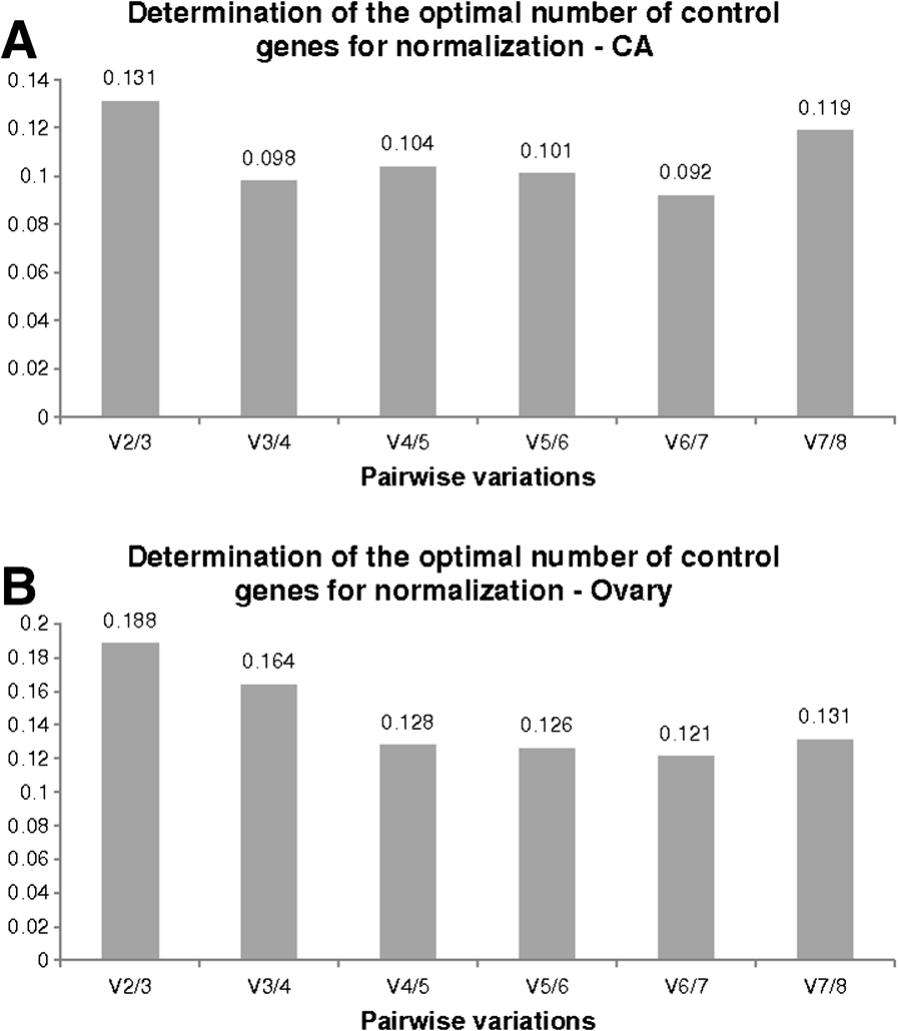
**Optimal number of reference genes for normalization as calculated by geNorm.** Pairwise variation analysis determining the optimal number of reference genes required ensuring accurate normalization between normalization factors NF_n_ and NF_n+1_ in **(A)** CA and **(B)** ovary during the gonadotrophic cycle.

In addition, geNorm can also determine the optimal number of genes required for accurate normalization. Upon inclusion of a less stable reference gene, geNorm calculates the pairwise variation (V) using sequential normalization factors (NFn and NFn + 1). The algorithm can thus determine if addition of the extra reference gene will add to the stability of the normalization factor. A cut-off value of V = 0.15 is recommended in deciding the addition of the next reference gene. In the CA, the lowest V-value was calculated following inclusion of the 7th most stable gene, this means that addition of the least stable gene *AnnIX* would negatively impact the normalization process. This is similar in the ovary, in which inclusion of *Actin* would result in a poorer normalization. Our results indicate that in CA, only two reference genes are necessary for accurate normalization since the V2/3-value was found to be below the cut-off of 0.15 (Figure 5A). In the ovary however, addition of the third most stable gene (*EF1a*) is needed, but adding a fourth is not imperative (Figure 5B).

The Normfinder analysis is based on an ANOVA (analysis of variance) model. This algorithm determines the genes with the least variation in expression over the whole sample set and can, if applicable, consider systematic differences between sample subgroups. During the gonadotrophic cycle of *D. punctata*, Normfinder ranked the eight reference genes in the CA as follows: *EF1a* >*Arm* >*SDHa* >*Actin* >*Tub* >*RpL32* >*GAPDH* >*AnnIX* (Figure 3B), with *EF1a* being the most stable with a stability value of 0.153 and *AnnIX* being the least stable with a stability value of 0.647. Calculation of stability values in the ovary resulted in: *EF1a* >*Tub* >*Arm* >*SDHa* >*RpL32* > *AnnIX* >*GAPDH* >*Actin*, with stability values for the best and the worst reference genes as 0.228 and 0.710 for *EF1a* and *Actin* respectively (Figure 4B).

#### Gene expression of target genes

**Figure 6:**
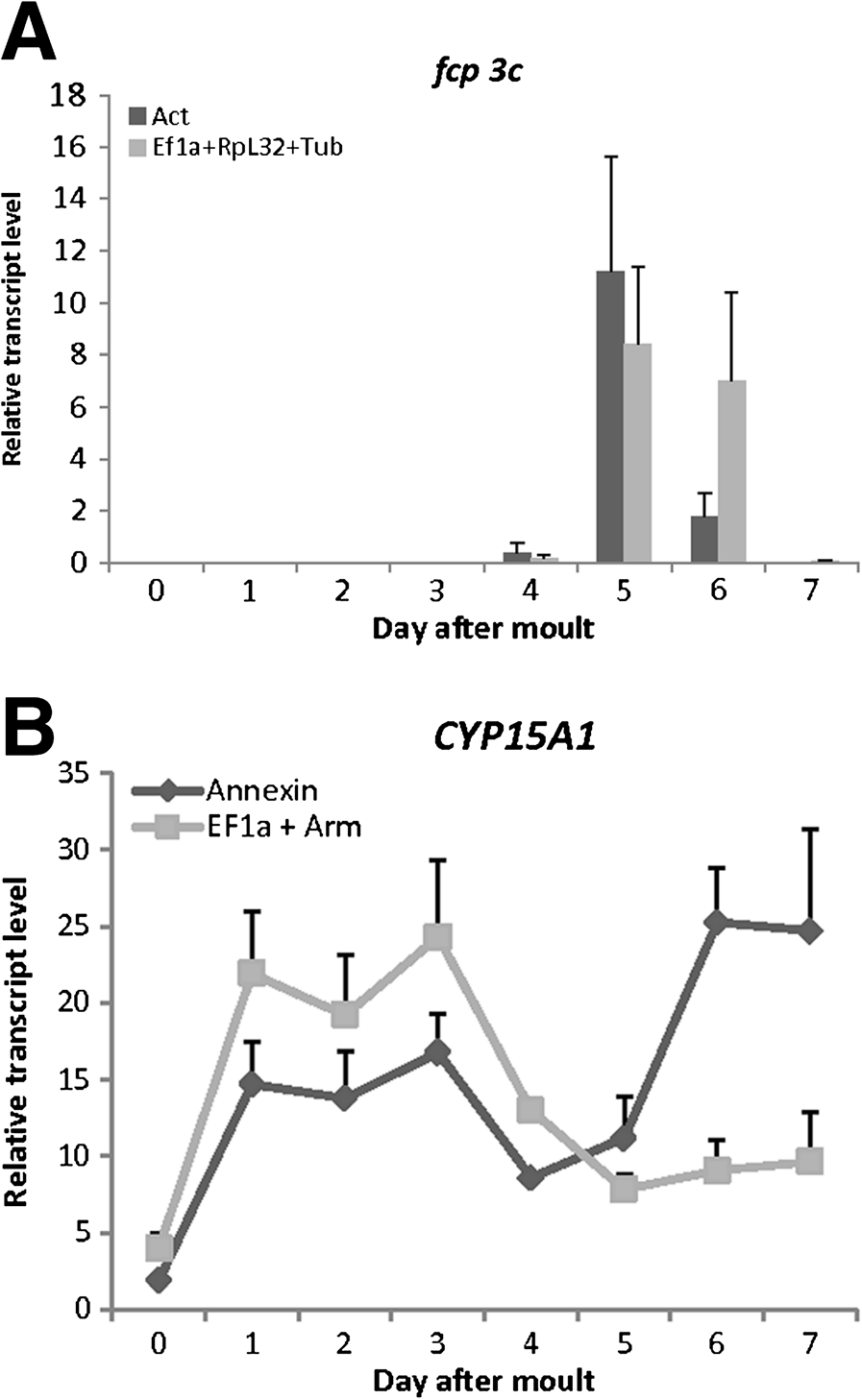
**Graphical representation of the relative transcript level of the target gene A) *follicle cell protein 3c *measured in the ovary and B) *CYP15A1 *in the CA during the first gonadotrophic cycle of *D. punctata*.** Measurements were taken every day during the cycle (day 0 to day 7 after final molt). The data represent means of three independent pools of ten animals, run in triplicate using q-RT-PCR. *Fcp 3c* was normalized to *Tub*, *RpL32* and *EF1*α transcript levels or to *Actin* transcript levels. *CYP15A1* was normalized to *EF1*α and *Arm* transcript levels or to *AnnIX* transcript levels. Vertical bars indicate S.E.M.

The selected set of reliable reference genes was used to analyze the relative expression of two target genes, *follicle cell protein 3c* and *CYP15A1* in the ovary and CA, respectively, during the first gonadotrophic cycle of *D. punctata*. As a proof of principle, the expression of both genes was measured using the recommended set of reference genes, as well as the gene that was determined to be the worst for normalization. The relative expression profile of *follicle cell protein 3c* is shown in Figure 6A. The results obtained by using *Tub*, *EF1a* and *RpL32* as reference genes and their in-run PCR efficiency estimates show a high expression of *fcp 3c* on days 5 and 6. However when the worst housekeeping gene, *Actin* was used to normalize, the relative expression on day 6 was found to be lower than when normalizing with the recommended set of reference genes.

The relative mRNA quantity of *CYP15A1* normalized to the recommended set of housekeeping genes (*EF1a* and *Arm*) and to only *AnnIX* is shown in Figure 6B. When performing normalization with *EF1a* and *Arm* the expression of *CYP15A1* correlates with JH biosynthesis. However, when only normalized to *AnnIX*, *CYP15A1* expression increases at the end of the gonadotrophic cycle when JH biosynthesis is low (Figure 1).

## Discussion

The sensitivity and range of q-RT-PCR has ensured that this technique is the most important in relative quantification of mRNA levels. However, the accuracy of q-RT-PCR studies using an internal control for normalization, is dependent on the reference genes used. The expression stability of these genes may be influenced by differences in the studied tissues, experimental treatments or physiological state. Therefore, the use of such genes without previous and proper validation for the experimental setup, can lead to the calculation of inaccurate expression levels of target genes and consequently to incorrect data interpretation [6]. To avoid bias from normalization, the use of multiple stably expressed references genes has become the gold standard in q-RT-PCR studies [29]. To our knowledge, a validation study of housekeeping genes for the relative quantification of mRNA has not been performed before in cockroaches. Unfortunately, to date, very limited sequence information is available for the Pacific beetle cockroach, *D. punctata*. Nevertheless, this animal is an appropriate model for endocrine studies of the reproductive cycle in primitive insects. This is especially true in studying the production and regulation of JH. To gain more insight into these regulatory processes up- and downstream of JH, q-RT-PCR was established for use as a robust technique to determine relative expression levels of target genes involved in the regulation of reproduction in this viviparous cockroach.

Eight possible reference genes were selected based on their traditional use and stability described in reported validation studies in other insects [10,12,13,16,30,31]. Next to the eight genes described in this study, one other gene widely used as a reference in other organisms, *ubiquitin*, was investigated. However, for the partial sequence that was picked up, the multiple q-RT-PCR primer pairs tested, did not result in single-band amplicons. The eight genes that were further investigated showed a relatively stable expression profile, characterised by AESM values far below the 1.5 cut-off value. Two tissues, CA and ovary, important in studying reproductive physiology, were chosen in this study. The stability of the eight genes during female development was followed from day 0 to day 7 after the imaginal molt. Both geNorm and Normfinder algorithms identified *Arm*, *SDHa* and *EF1a* as the most stable reference genes in the CA. By far the least stable was shown to be *AnnIX*. In the ovary, geNorm identified *RpL32*, *Tub* and *EF1a* as the most stable, whereas the Normfinder analysis showed *Tub*, *EF1a* and *Arm* to be the most stably expressed. The consensus is that *Tub* and *EF1a* can be used to normalize the expression of target genes in the ovary. However, since Figure 5B indicates that a third gene is necessary to ensure accurate normalization, the geometric mean of *Tub*, *EF1a* and *RpL32* was used to normalize the expression of the target gene *follicle cell protein 3c* (Figure 6A). Differences in the outcome of both algorithms have been observed before [13,16,32,33] and can be explained by their calculation methods. Whereas geNorm eliminates the least stable gene in a stepwise manner and recalculates the AESM scores at each step, Normfinder follows a model-based approach, taking into account all the reference genes at one time. Moreover, geNorm assumes that the expression ratios of two ideal reference genes are identical in all conditions. The program is therefore highly sensitive to co-regulation, selecting genes with high degrees of similarity in their expression profiles. This disadvantage of geNorm was avoided by choosing candidate reference genes involved in different processes in cell housekeeping (Table 1). Other useful software, Bestkeeper, can be used to validate reference genes [9]. However, because this algorithm is similar to geNorm, its use was not included in this study.

Figure 5 shows that a large drop is seen in the V-value upon inclusion of a third or fourth reference gene for normalization in the CA or ovary respectively. A large drop in V-value indicates a more accurate normalization with addition of the next best reference gene. However, since the V2/3 and V3/4 values in CA and ovary, respectively, are below the cut-off of 0.15, the practicality and cost of using more reference genes in the q-RT-PCR setup must be considered.

As a proof of principle, our validation results were tested by measuring the expression of two target genes in the ovary. *Follicle cell protein 3c* was found to be selectively expressed in follicle cells during the period of vitelline membrane formation in *Drosophila melanogaster*[34]. A very elegant study in the German cockroach, *Blattella germanica* designed a suppression subtractive hybridization library of the ovary aimed at identifying genes specifically expressed after vitellogenesis [27]. In this library, an ortholog of *follicle cell protein 3C* was found. Its expression pattern showed a role in chorion formation. *CYP15A1* was first functionally characterized in *D. punctata*. It encodes a cytochrome P450 enzyme catalyzing the epoxidation of methyl farnesoate to JH [26]. The gonadotrophic cycle has been well described in *D. punctata*. Mating of adult females on day 0 stimulates enhanced JH biosynthesis [35]. During the subsequent gonadotrophic cycle, both JH production and oocyte length rapidly increase, then as vitellin accumulates in the oocytes, JH synthesis declines on day 5. After vitellin content reaches a maximum, the chorion forms and ovulation occurs on day 7. At this time, the rate of JH production falls (as seen in Figure 1) and remains low during the gestation period [19,21]. The structure-activity relationship of the CA during the first gonadotrophic cycle of *D. punctata* has been described in great detail. A clear correlation exists between volume of CA cells, the number of cells per CA and the fine structure of organelles on the one hand and the JH biosynthetic capacity on the other. This increase in JH biosynthetic activity is not caused by an increase in cell number, but by an increase in the biosynthetic capacity per cell [36-38]. The expression pattern of *Diploptera follicle cell protein 3c* as shown in Figure 6A is consistent with the involvement of this gene in chorion formation, as earlier described for *Blattella* and *Drosophila*. When only normalized to *Actin*, the expression of *fcp 3c* on day 6 drops relative to values normalized with the recommended set of reference genes; even though, on this day, chorion formation is still in full progress. In the case of *CYP15A1*, the expression rises drastically towards the end of the gonadotrophic cycle when only normalized to *AnnIX* (Figure 6B). However, when normalizing with the recommended set of genes, *CYP15A1* expression correlates well with the JH biosynthetic activity in the CA, as previously reported [26].

## Conclusion

This study provides the first validation of reference genes for q-RT-PCR studies in cockroaches. Eight possible reference genes with relatively stable expression have been identified. Follow-up studies investigating relative mRNA levels in differing experimental setups will therefore become more straightforward. These results clearly show that some conventionally used reference genes such as *ß-actin* may not be the best choice for normalization of gene expression in cockroaches and that it is important to always confirm the stability of reference genes in the specific system and experimental setup of interest.

## Methods

### Animals

The *D. punctata* colony was maintained at 27°C in constant darkness and animals were fed lab chow and water *at libitum*. To obtain pools of synchronised animals, newly molted female adult cockroaches were picked from the colony, placed in separate containers and provided with water and lab chow for the next 7 days. Mated status was confirmed by the presence of a spermatophore.

### Sample collection

*D. punctata* adult females were dissected in cockroach ringer solution (150 mM NaCl, 12 mM KCl, 10 mM CaCl_2_.2H2O, 3 mM MgCl_2_.6H2O, 10 mM HEPES, 40 mM Glucose, pH 7.2) using a dissecting microscope. For each dissected animal, oocyte lengths were measured. CA and ovary samples were taken from day 0 to 7 of adult female development. Attached fat body was removed from tissues in sterile saline. Ovary and CA samples were flash-frozen in liquid nitrogen to prevent RNA degradation and were stored at −80°C until further processing. For each time point, three biologically independent pools of 10 animals each were collected.

### RNA extraction and cDNA synthesis

Pooled ovary samples were homogenised with RNase-free pestles and total RNA was extracted using the RNeasy Lipid Tissue Kit (Qiagen) according to the manufacturer’s instructions. An additional DNase treatment (RNase-free DNase set, Qiagen) was performed to eliminate potential genomic DNA contamination. Because of the small size of the CA, RNA from this tissue was extracted using the RNAqueous^®^-Micro Kit (Ambion), followed by the recommended DNase step. Quality and concentration of the resulting RNA samples were measured using a Nanodrop spectrophotometer (Thermo Scientific). Only intact RNA was used in subsequent PCR reactions. An equal amount of RNA (500 ng for ovary, 200 ng for CA) was transcribed in subsequent cDNA synthesis utilising Superscript III and random hexamers in a final volume of 20 μl following the manufacturer’s protocol (Invitrogen Life Technologies). All samples were reverse transcribed together in a single run. The resulting cDNA samples were diluted 10-fold with PCR grade water. A calibrator sample was prepared by pooling 5 μl of each cDNA sample. In the same run, negative control reactions were set up without reverse transcriptase enzyme to test for genomic DNA contamination.

### Radiochemical assay (RCA)

Rates of JH release were measured using the *in vitro* radiochemical assay (RCA) originally described by Tobe & Pratt [39] and Pratt & Tobe [40]. Day 0 to day 7 female cockroaches were immobilized on ice and their oocytes measured. CA were dissected and transferred to TC199 medium in a sterile environment. A short-term *in vitro* assay of JH release in TC199 medium (GIBCO) [2% Ficoll, 1.3 mM CaCl_2_ · 2H_2_O and 3 μCi/ml l-[*methyl*-^14^C]methionine (55 mCi/mmol; American Radiolabeled Chemicals, Inc.) followed by rapid partition was conducted on individual corpora allata according to Feyereisen and Tobe [41] and Tobe and Clarke [42]. JH release was measured from 12 individual CA isolated from 12 different animals (n = 12).

### Sequencing of candidate reference genes

Since no genome or full transcriptome sequence data is currently available for *D. punctata*, a first set of degenerate primers was developed based on a multiple sequence alignment of known orthologous sequences from different insects. Such an alignment was made for EF1-α, GAPDH, armadillo, SDH, annexin, tubulin, RpL32 and for the target follicle cell protein 3c. For ß-actin, a *Blattella germanica* sequence is known and was used to design specific primers. Partial sequences for these genes were obtained with the primers listed in Table 1. A temperature-gradient polymerase chain reaction (PCR) was run using Taq DNA polymerase (Sigma-Aldrich Co.) with either *D. punctata* day 6 ovary or day 4 embryo cDNA as a template. In this PCR, the following thermocycling profile was used: 3 min at 95°C followed by 30 cycles of 30 s at 94°C, 1 min at 55°C (with a 10°C gradient) and 1 min at 72°C. The amplification products were analyzed after a horizontal agarose gel electrophoresis and visualized using UV. Bands of the expected size were cut out and further purified using the GenElute™ Gel extraction Kit (Sigma-Aldrich Co.). The resulting DNA fragments were subcloned into a CloneJET™ cloning vector using the CloneJET™ PCR Cloning Kit (Fermentas) and sequenced.

### Quantitative real-time PCR (q-RT-PCR)

q-RT-PCR primers were designed using Primer Express software (Applied Biosystems) (Table 2). Primer sets were validated by designing relative standard curves for gene transcripts with serial 10-fold dilutions of the calibrator’s cDNA sample. Efficiency of q-RT-PCR and correlation coefficient (R^2^) were measured for each primer pair.

q-RT-PCR reactions were performed in triplicate on a CFX384 Touch™ Real-Time PCR Detection System (Bio-Rad) in a 10 μl volume containing 5 μl IQ™ SYBR® Green Supermix (Bio-Rad), 1 μl forward and reverse primer (5 μM), 2 μl of MQ-water and 1 μl of cDNA. A two-step thermal cycling profile was used: 95°C for 3 min, followed by 40 cycles of 95°C for 10 s and 59°C for 30 s. Upon completion of every run, a dissociation protocol (melt curve analysis) was performed to check for primer dimers. Additionally, a few representative PCR products were run on a 1.2% agarose gel containing GelRed™ (Biotium). After electrophoresis only a single band could be seen which was further cloned and sequenced (CloneJET™ PCR Cloning Kit, Fermentas) to confirm target specificity.

Three biologically independent pools (each containing CA/ovary from 10 different animals) taken at each time point measured, were used to obtain the described results.

### Gene stability analysis

The quantitative expression measurements relative to the calibrator sample of all the candidate reference genes tested in either CA or ovary during the gonadotrophic cycle were analyzed using the available normalization programs geNorm [6] and Normfinder [4], available from the website of the authors: http://medgen.ugent.be/~jvdesomp/genorm/ and http://www.mdl.dk/publicationsnormfinder.htm, respectively. Relative mRNA levels of *follicle cell protein 3c* and *CYP15A1* were normalized to the geometric mean of the relative quantity values for the required amount of most stably expressed reference genes selected by geNorm and NormFinder or to the relative quantity of the worst reference gene and relative to the calibrator sample [6].

## Available supporting data

The sequence information on the genes described in this paper is available in NCBI GenBank (http://www.ncbi.nlm.nih.gov/genbank/).

## Competing interests

The authors declare that they have no competing interests.

## Authors’ contributions

This study was conceived and coordinated by EM and SST. EM, EFH and JH designed the experimental protocol. JH, EM and EFH reared the cockroaches and performed the dissections. JH performed all RNA extractions and RCA. JH and EM prepared cDNA samples. EM and EFH selected the reference genes, cloned and sequenced PCR products for the eight candidate genes, designed and tested primers for q-RT-PCR, performed the q-RT-PCRs and carried out the analyses. EM wrote the manuscript with help from EFH, JH and SST; all authors read and approved the final manuscript.

## Supplementary Material

Additional file 1 **Partial sequences of the eight candidate reference genes cloned from *D. punctata.*** Q-RT-PCR primers are indicated in italics and underlined. Click here for file

Additional file 2 Raw data of the q-RT-PCR assays for CA and ovary. Click here for file
