# Supplementary material for: Sequencing and validation of housekeeping genes for quantitative real-time PCR during the gonadotrophic cycle of Diploptera punctata
Source: BMC Res Notes. 2013 Jun 19;6:237. doi: 10.1186/1756-0500-6-237 (PMC3750588; doi:10.1186/1756-0500-6-237)
Supplement: Additional file 1 — Partial sequences of the eight candidate reference genes cloned from D. punctata. Q-RT-PCR primers are indicated in italics and underlined. [file 1756-0500-6-237-S1.docx]

**Additional file 1**

Partial sequences of the eight candidate reference genes cloned from *D. punctata*. Q-RT-PCR primers are indicated in italics and underlined.

>DippuActin

CATCAGGGAGTCATGGTCGGCATGGGCCAGAAAGACAGCTATGTTGGAGATGAGGCCCAGTCCAAGAGAGGTATCCTCACCTTGAAATATCCTATTGAGCACGGAATCGTAACCAACTGGGATGACATGGAAAAAATCTGGCATCATACCTTCTACAATGAACTTCGAGTTGCTCCCGAAGAACATCCTGTGCTTCTTACCGAAGCCCCCCTTAACCCAAAGGCTAACAGAGAAAAGATGACACAGATCATGTTTGAAACTTTCAACACCCCTGCGATGTACGTAGCTATCCAGGCTGTGCTGTCACTATATGCCTCTGGTAGAACCACTGGTATTGTGCTGGATTCAGGCGATGGTGTC*TCGCACACAGTACCAATCTATGAA*GGTTATGCTCTGCCCCATGCCATCCTGCGTCTG*GATCTGGCTGGTCGTGACTTG*ACTGACTACCTCATGAAGATCCTGACCGAGCGTGGCTACAGTTTCACAACTACAGCTGAGAGAGAAATTGTCAGAGACATCAAAGAAAAACTTTGCTATGTTGCTCTTGACTTTGAACAAGAAATGGCTACTGCTGCATCCTCCAGCTCATTGGAGAAGTCATATGAACTTCCTGACGGTCAGGTAATCACCATTGGAAACGAAAGGTTCCGTTGTCCTGAAGCTCTGTTCCAACCTTCATTCTTGGGAATGGAAGCCTGCGGTATCCACGAGACAACATACAATTCCATCATGAAGTGTGATGTGGACATCCGTAAAGACCTGTAT

>DippuEF1a

TTCGAGAAGGAAGCCCAGGAAATGGGCAAAGGTTCCTTCAAGTATGCTTGGGTTTTGGACAAACTGAAGGCTGAACGTGAACGTGGTATTACAATTGATATTGCATTGTGGAAGTTTGAAACTAGTAAATACTATGTAACAATTATTGATGCTCCTGGACACAGAGATTTCATCAAGAACATGATCACAGGAACCTCTCAGGCTGACTGTGCGGTACTTATTGTTGCTGCCGGTACTGGTGAATTTGAAGCTGGTATTTCCAAGAATGGTCAGACTCGTGAGCATGCTCTTCTGGCTTTCACTTTGGGTGTAAAGCAACTGATTGTAGGTGTAAACAAAATGGACTCAACTGAACCACCATACAGTGAAACTCGTTTTGAGGAAATCAAGAAGGAAGTTAGTAACTACATTAAGAAGATTGGTTACAATCCTGCTGCTGTAGCATTTGTGCCAATCTCTGGCTGGCACGGAGACAACATGTTGGAACATTCCGACAAGATGTCATGGTTTAAGGGTTGGTCTATTGATCGTAAGGAGGGCAAGGCTGAAGGAAAATGTCTGATTGAGGCTTTGGATGCTATTCTTCCACCTAGCCGACCAACGGAGAAGCCCCT*TCGTCTTCCTCTGCAGGATGTCT*ACAAGATTGGAGGTATTGGAACAGTGCCTGTTGGCAGAGTTGAAACTGGTGTGTTGAAACCA*GGTATGGTTGTGACATTTGCACCC*GCCAACTTGACCACTGAAGTAAAGTCTGTTGAAATGCACCATGAAGCTCTTCAAGAAGCTGTTCCTGGAGACAACGTTGGCTTCAACGTTAAGAACGTTTCTGTTAAGGAACTGCGT

CGTGGCTACGTTGCTGGTGACTCTAAGAACAACCCCCCTAGAGGAGCTGCTGACTTTATTGCTCAGGTTATTGTGTTGAATCACCCTGGTCAGATTTCAAATGGCTACACTCCAGTACTAGACTGTCACACAGCTCATATTGCTTGCAAGTTTGCCGAGATCAAGGAAAAGTGCGACCGTCGTACAGGCAAAACAACTGAGGAGAATCCAAAGTCTATCAAGTCTGGTGATGCTGCCATAGTAAATCTTGTTCCCAGCAAACCAATGTGCGTGGAATCTTTCCAGGAATTCCCACCTCTTGGGCGTTTTGCTGTGCGTGACATGAGACAGACTGTTGCAGTCGGTGTCATTAAGAGTGTTACTGCTAAGGAAGCTACAAGTGGTAAGGTAACGAAGGCTGCTGAGAAGGCCCAGAAGAAGAAATAA

>DippuGAPDH

ATGTCAAAGATTGGAATCAACGGATTTGGCCGTATTGGTCGTCTCGTTCTCAGAGCAGCCCTAGAAAAGGGAGCACAGGTTGTTGCTATCAACGACCCATTTATTGGTTTGGACTACATGGTGTACATGTTCAAATATGACTCAACTCACGGTAGATTCAAAGGCGAAGTGAAAGCTGAGGGAGATCAACTCGTTGTGAATGGCCAGAAGATTGCTGTTTTTGCGGAGAGAGATCCCAAGGCCATTCCATGGGGAAAAGCTGGTGCAGATTATGTAGTGGAATCCACTGGTGTTTTCACAACCATTGACAAGGCATCGGCTCACTTGGAAGGTGGTGCTAAGAAGGTAATCATCTCAGCGCCCAGTGCAGACGCACCTATGTTTGTTGTTGGTGTGAATCATGAAACATATGATCCATCACTTAAGGTTGTTTCCAACGCTTCCTGCACCACCAACTGCTTGGCTCCACTTGCCAAGGTCATTCACGATAATTTTGAGATTGTGGAAGGTCTTATGACAACAGTTCATGCAGTCACTGCAACACAGAAGACAGTAGACGGACCTTCTGGAAAGTTGTGGCGTGATGGACGTGGTGCTGCACAAAACATTATCCCAGCTTCAACTGGTGCAGCAAAAGCTGTAGGCAAAGTCATTCCTGCACTTAATGGAAAGCTGACTGGCATGGCTT*TCCGTGTGCCTGTTCCTAATG*TCTCAGTCGTAGACCTCACTG*TCAGACTTGGCAAGGCAGC*AACGTACGATGAAATCAAAGCCAAGGTGAAGGAAGCATCTCAAGGACCAATGAAGGGAATTTTGGACTACACTGAAGACGATGTTGTATCATCTGACTTCATCGGCGATAACCACTCCTCCATTTTTGATGCAAAGGCTGGAATTCCTCTCAATAATAATTTTGTGAAACTCATTTCATGGTATGACAATGAATTTGG

>DippuArm

ATGGAGGAGATAGTGGAGGGAACAGTTGGAGCTCTCCACATTCTGGCTAGAGAGTCAAATAATCGTGCCATAATTAGATCACAGCTGGTCATTCCTATATTTGTGCAGTTGTTATTTAATGAGATTGAGAACATTCAGCGAGTAGCAGCTGGAGTTCTCTGTGAGTTGGCTGCTGATAAAGAAGGAGCAGAGATGATAGAACAGGAAGGA*GCTACTGCACCACTCACAGAATTATTA*CATTCACGAAATGAAGG*TGTTGCAACGTATGCTGCAG*CAGTTCTCTTCCGAATGTCTGAAGACAAGCCCCAAGACTACAA

>DippuRPL32

GTGTACAGACCTAAAATTGTCAAGAAGAGAACTAAGCATTTTATCCGCCATCAGAGTGACCGCTACAAGAAATTGAAGAAGTCATGGAGGAAGCCTAAAGGTATTGACAACAGGGTGCGAAG*GCGCTTCAAGGGCCAGTAC*CTGATGCCATCTATCGGTTA*TGGTAGCAATAAGAAAACCAAGCA*CATGCTGCCAACTGGTTTCAGGAAGGTTTTGGTACACAATGTGAAGGAACTTGAAATCCTCATGATGCAGAACCGCAAATACTGC

>DippuSDHa

GCAGATTGGCAGTGGCACATGTATGACACAGTGAAAGGTTCTGACTGGCTTGGAGATCAGGATGCTATCCACTATATGACAAGGGAAGCACCAAAAGCTGTTGTTGAACTGGAAAATTATGGAATGCCTTTCAGCAGAACGCAGGATGGTAAAATCTATCAGAGAGCATTTGGTGGACAGTCGCTGAAGTTTGGTAAAGGTGGTCAGGCTCACAGGTGCTGCTGTGTGGCCGATCGTACGGGTCACTCCCTCCTGCACACTCTGTATGGACAGTCCCTGCGCTATGACTGTAACTACTTTGTGGAGTACTTCGCCCTTGACCTTCTTATGGAAGAGGGAGAGTGCCGTGGTGTCATTGCCTTATGTCTAGAAGACGGTAGCATTCACAGATTCCACTCCAAGAGCACGGTGTTGGCGACAGGTGGGTATGGCCGAGCTTACTTCTCATGCACATCTGCACACACTTCGACAGGAGATGGTACTGCCATGGTGTCTAGAGCAGGCCTACATAACGAAGACTTGGAGTTTGTTCAGTTTCATCCTACAGGTATATATGGAGCAGGCTGCCTGATGACAGAAGGATGTCGAGGTGAAGGTGGTTACCTGATAAACAGCGAGGGTGAGAGGTTTATGGAACGTTATGCTCCTGTAGCAAAGGACTTGGCTTCACGAGACGTAGTGTCCAGATCTATGACAATTGAAATTAGGGAAGGAAGAGGTTGTGGACCTGAGAAGGACCATGTATTCCTGCAGTTGCACCACTTGCCACCAGAGCAGCTAGCCACAAGGTTACCCGGTATCTCAGAGACAGCCATGATCTTTGCTGGTGTGGATGTGACACGAGAGCCTATCCCAGTCCTACCAACTGTGCACTATAACATGGGAGGAGTTCCTACCAACTATAGAGGACAGGTGTTGACGGTGTCTGGAGGACAAGATAAGGTCGTTCCTGGTCTGTATGCCTGTGGAGAGGCAGCAT*GCTCTTCTGTGCATGGTGCTAA*CAGGCTCGGTGCCAACTCTCTGCTTGAT*CTTGTTGTGTTCGGACGTGC*CTGTGCCAAGACCATTGCAGAGGAACACAAACCTGGCGAGACCATTGGATCACTCAAACCTAGTTCTGGAGAAATTTCAGTTGCAAACTTAGACAAACTGCGTCATGCTGATGGTTCAGTATCGACTGCTGCTCTACGTCTGAGCATGC

>DippuAnnIX

ACCCAGCTGAGCCTTTTGACTCAAATGCAGATGCAGAAATGCTGAGAGCTGCTATGAAAGGTTTTGGAACAGATGAGCAGGTAATAGTCGACATTCTGTCCAAGCGTGGTGTGGTG*CAGAGGTTGGAGATTGCTGA*GACATTCAAGACACTTTATGGAAAGGATCTTGTGAAGGAGTTGAAAAGTGAACTGG*GAGGAGCATTTGAAGATGCA*GTGGTCTCCCTTATGACATCACTTCCTGATCTCTATGCTAAGGAACTTCATGATGCTATCAGTGGGATTGGTACAGACGAAGAAGCCATTGTTGAAATACTCTGCACTCTCAGCAACTATGGTCTTCGTACAATAACAGCTGTTTATGAAAAATTGTATGAAAGTCCTTTGGAGAGTGATTTGAAAGGAGACACATCCGGCCACTTCAAGAGGCTACTTGTTTCACTCCTTACTGCAAACAGAGATGAGAGTTATGAAGTGGACAAAGCAGAAGCCATTGCCGATGCGGAATCATTGATGGCAGCAGGGGAAATGCAGTGGGGAACTGATGAATCTGTATTCAACTCCGTCCTTGTGTCTAGAAGTTACGTCCAGTTACGTCATATCTTCAGGGAATATGAGAAACTCGCTGGACATGATATTGAGACAGCCATTAAACGTGAATTTGCTGGCAGTTTGGAAGATGGCTATTTGTCTATTGTGAAATCTGTGAAGAATAAAACAGGGTACTTTGCTGAGAGGCTACACAATGCAATGGCTGGGATGGGAACAAAAGACAGAACTCTGATCCGTATAATTGTAACTCGCTCCGAGATCGACCTGGCGGACATCAA

>DippuTub

ATGCGTGAGTGTATCTCGGTGCACGTTGGTCAGGCTGGTGTGCAGATCGGCAATGCATGCTGGGAACTCTACTGCCTGGAACATGGCATCCAACCTGATGGTCAGATGCCATCAGATAAGACATTGGGAGGTGGTGATGACAGTTTCAACACATTTTTCAGCGAGACTGGTGCAGGAAAGCATGTCCCAAGGGCTGTTTTTGTGGATTTGGAACCTACTGTAGTTGATGAGGTGCGTACTGGTACATATCGGCAGCTATTCCACCCTGAACAGCTAATCACTGGCAAAGAAGATGCTGCCAACAATTATGCACGAGGTCACTACACGATTGGCAAGGAAATTGTTGACCTTGTTTTGGACCGCATTAGGAAATTGGCAGATCAATGTACAGGATTGCAAGGTTTCCTCATCTTCCACTCATTTGGTGGAGGTACTGGTTCTGGATTCACCTCTCTGTTGATGGAACGGCTATCAGTTGACTACGGCAAGAAGAGTAAATTGGAATTTGCTATTTACCCTGCTCCACAAGTTTCCACTGCAGTTGTAGAACCCTACAACTCCATCCTGACCACACATACCACTCTGGAACATTCCGACTGCGCCTTCATGGTAGACAATGAAGCTATTTATGATATCTGCAGACGAAACTTGGATATTGAACGCCCAACCTACACGAACCTCAACAGACTTATTGGCCAAATTGTATCCTCAATCACTGCCTCCCTTAGATTTGATGGTGCCCTCAATGTTGATCTGACTGAATTCCAAACAAACTTGGTTCCATACCCACGAATCCATTTCCCTCTTGTAACTTATGCACCTGTTATCTCTGCAGAAAAGGCTTACCATGAGCAACTCTCTGTAGCAG*AAATTACCAACGCTTGCTTTGAA*CCTGCAAACCAAATGGT*AAAATGCGATCCTCGCCA*TGGAAAATACATGGCTTGCTGTATGTTGTACAGAGGAGACGTCGTGCCAAAGGATGTAAATGCTGCCATCGCCACCATCAAAACAAAGCGAACAATTCAGTTCGTGGACTGGTGTCCCACTGGTTTCAAGGTTGGTATTAATTACCAGCCTCCCACTGTTGTTCCTGGTGGAGATTTGGCCAAGGTGCAACGTGCAGTTTGCATGTTGTCCAACACTACAGCAATTGCTGAGGCCTGGGCTCGTCTGGACCACAAGTTTGATCTGATGTATGCAAAACGTGCTTTTGTCCACTGGTATGTAGGAGAGGGTATGGAAGAAGGAGAATTCTCTGAAGCTCGGGAGGATCTTGCTGCCCTCGAGAAAGACTACGAGGAAGTCGGCATGGACTC

>Dippu follicle cell protein 3c

GTGGAGTGTTTTTGAGTGGTCAGTTCGTCAAGGGAAGTCCAGAACCTCCGAAAGGAAATGCTGTGGTGCTTCATGAACTTATGGAAAA*TCTACCTTGCAACGCTTTTG*GCAACAAACAGTGCACAAATAAGTGCCTTGACAGTATTGTGAAATATCTACCGAACAGTCCTGCTCTAGTG*TGTGGCTCAATAGACAGGGA*CTGCTACAAAGAAAGAGCATATCTGTTCATCAAGAACTGTAGCGACACGTGGGTCAACACAAACATGTCAGCAGGACGGGAATACTGCTG
