# Supplementary material for: Sequencing and validation of housekeeping genes for quantitative real-time PCR during the gonadotrophic cycle of Diploptera punctata
Source: BMC Res Notes. 2013 Jun 19;6:237. doi: 10.1186/1756-0500-6-237 (PMC3750588; doi:10.1186/1756-0500-6-237)
Supplement: Additional file 2 — Raw data of the q-RT-PCR assays for CA and ovary. [file 1756-0500-6-237-S2.docx]

**Additional file 2** Raw data of the q-RT-PCR assays for CA and ovary.

| **CA** | Annexin | Actin | Armadillo | Sdh | EF1alfa | GAPDH | Tubulin | RP49 |
| --- | --- | --- | --- | --- | --- | --- | --- | --- |
| D0 | 25.50 | 23.53 | 29.26 | 26.97 | 21.93 | 27.82 | 23.96 | 25.65 |
| D1 | 26.20 | 24.52 | 29.52 | 27.35 | 22.12 | 26.82 | 24.27 | 25.29 |
| D2 | 26.22 | 23.78 | 29.71 | 27.51 | 21.77 | 26.22 | 23.50 | 25.21 |
| D3 | 27.20 | 27.20 | 30.62 | 27.83 | 22.76 | 27.27 | 25.11 | 25.99 |
| D4 | 26.25 | 23.67 | 30.14 | 27.44 | 21.63 | 26.50 | 24.43 | 25.07 |
| D5 | 25.85 | 21.98 | 28.59 | 26.43 | 20.33 | 25.23 | 24.08 | 24.34 |
| D6 | 28.80 | 24.04 | 30.30 | 28.41 | 22.01 | 26.56 | 25.44 | 25.34 |
| D7 | 28.37 | 24.13 | 30.01 | 28.40 | 21.75 | 26.29 | 25.58 | 25.08 |
|  |  |  |  |  |  |  |  |  |
| **Ovary** | Actin | EF1a | GAPDH | Arm | RpL32 | SDHa | AnnIX | Tub |
| D0 | 21.64 | 20.73 | 24.80 | 26.80 | 24.22 | 26.20 | 23.15 | 22.20 |
| D1 | 22.13 | 20.86 | 24.12 | 27.70 | 24.05 | 26.53 | 24.28 | 21.89 |
| D2 | 20.81 | 20.22 | 23.50 | 27.00 | 23.26 | 26.38 | 23.97 | 21.90 |
| D3 | 22.14 | 19.86 | 22.42 | 27.16 | 22.16 | 26.28 | 24.56 | 20.56 |
| D4 | 20.96 | 18.83 | 21.60 | 26.30 | 21.50 | 25.98 | 23.31 | 19.98 |
| D5 | 21.65 | 20.16 | 24.06 | 27.02 | 22.62 | 26.86 | 24.67 | 21.38 |
| D6 | 19.01 | 20.27 | 24.91 | 25.84 | 23.27 | 27.21 | 23.34 | 21.37 |
| D7 | 20.54 | 21.49 | 24.10 | 26.35 | 23.88 | 26.94 | 22.57 | 20.65 |
